# Phenolic Content, Antioxidant, Antibacterial, Antihyperglycemic, and α-Amylase Inhibitory Activities of Aqueous Extract of *Salvia lavandulifolia* Vahl

**DOI:** 10.3390/ph16030395

**Published:** 2023-03-06

**Authors:** Firdaous Remok, Soukaina Saidi, Aman Allah Gourich, Khalid Zibouh, Mohamed Maouloua, Fadwa El Makhoukhi, Naoual El Menyiy, Hanane Touijer, Mohamed Bouhrim, Sevser Sahpaz, Ahmad Mohammad Salamatullah, Mohammed Bourhia, Touriya Zair

**Affiliations:** 1 Research Team of Chemistry of Bioactive Molecules and the Environment, Laboratory of Innovative Materials and Biotechnology of Natural Resources, Faculty of Sciences, Moulay Ismaïl University, Meknes 50070, Morocco; 2 Laboratory of Molecular Chemistry, Materials and Catalysis, Faculty of Science and Technology, Sultan Moulay Slimane University, Beni Mellal 23000, Morocco; 3 Laboratory of Medical Biology, Med V Provincial Medical Center, Meknes 50000, Morocco; 4 National Center for Scientific and Technical Research, Rabat 10000, Morocco; 5 Laboratory of Pharmacology, National Agency of Medicinal and Aromatic Plants, Taounate 34025, Morocco; 6 Joint Research Unit 1158 BioEcoAgro, INRAE, JUNIA, University of Lille, University of Liège, UPJV, University of Artois, ULCO, ICV—Institut Charles Viollette, 59000 Lille, France; 7 Department of Food Science & Nutrition, College of Food and Agricultural Sciences, King Saud University, Riyadh 11451, Saudi Arabia; 8 Laboratory of Chemistry and Biochemistry, Faculty of Medicine and Pharmacy, Ibn Zohr University, Laayoune 70000, Morocco

**Keywords:** *Salvia lavandilufolia* Vahl, diabetes, α-amylase, antioxidant, antimicrobial activity, quality control

## Abstract

*Salvia lavandulifolia* Vahl essential oil is becoming more popular as a cognitive enhancer and treatment for memory loss. It is high in natural antioxidants and has spasmolytic, antiseptic, analgesic, sedative, and anti-inflammatory properties. Its aqueous extract has hypoglycemic activity and is used to treat diabetic hyperglycemia, but few studies have focused on it. The objective of this work is to evaluate the various biological and pharmacological powers of *Salvia lavandulifolia* Vahl leaf aqueous extract. Quality control of the plant material was first carried out. Followed by a phytochemical study on the aqueous extract of *S. lavandulifolia* leaves, namely phytochemical screening and determination of total polyphenols, flavonoids, and condensed tannins contents. Then, the biological activities were undertaken, in particular the antioxidant activity (total antioxidant activity and trapping of the DPPH° radical) and the antimicrobial activity. The chemical composition of this extract was also determined by HPLC-MS-ESI. Finally, the inhibitory effect of the α-amylase enzyme as well as the antihyperglycaemic effect was evaluated in vivo in normal rats overloaded with starch or D-glucose. The aqueous extract obtained by use of the decoction of leaves of *S. lavandulifolia* contains 246.51 ± 1.69 mg EQ of gallic acid/g DE, 23.80 ± 0.12 mg EQ quercetin/g DE, and 2.46 ± 0.08 mg EQ catechin /g DE. Its total antioxidant capacity is around 527.03 ± 5.95 mg EQ of ascorbic acid/g DE. At a concentration of 5.81 ± 0.23 µg/mL, our extract was able to inhibit 50% of DPPH° radicals. Moreover, it showed bactericidal effect against *Proteus mirabilis*, fungicidal against *Aspergillus niger*, *Candida albicans*, *Candida tropicalis*, and *Saccharomyces cerevisiae*, and fungistatic against *Candida krusei*. A marked antihyperglycemic activity (AUC = 54.84 ± 4.88 g/L/h), as well as a significant inhibitory effect of α-amylase in vitro (IC_50_ = 0.99 ± 0.00 mg/mL) and in vivo (AUC = 51.94 ± 1.29 g/L/h), is recorded in our extract. Furthermore, its chemical composition reveals the presence of 37.03% rosmarinic acid, 7.84% quercetin rhamnose, 5.57% diosmetin-rutinoside, 5.51% catechin dimer, and 4.57% gallocatechin as major compounds. The antihyperglycemic and α-amylase inhibitory activities, associated with the antioxidant properties of *S. lavandulifolia*, justify its use in the treatment of diabetes in traditional medicine and highlight its potential introduction into antidiabetic drugs.

## 1. Introduction

Aromatic and medicinal plants have played a crucial role in the treatment and prevention of a wide range of diseases for thousands of years [1]. The therapeutic uses of herbs are based on plant chemistry [2]. Having an awareness of the chemical make-up of plants enables one to have a greater comprehension of the therapeutic potential of certain plants. Secondary metabolites, in particular, have been shown to have a variety of biological effects and have been labeled as antioxidants, antibiotics, antifungals, and antivirals and thus are capable of protecting plants from pathogens [3]. Sixty percent to eighty percent of the world’s population uses herbal medicine as its primary source of healthcare [4]. The Lamiaceae family is a plant family with a global distribution that includes approximately 236 genera (6900–7200 species). Salvia is the family’s largest genus, with nearly 900 species [5].

It is widespread in the tropics as well as in the temperate zones of the world in the following order: the Mediterranean basin, central Asia, the American continent, the Pacific islands, and equatorial Africa and China [6]. Species of this genus have shown many biological activities [7]. In traditional medicine, sage is one of the oldest medicinal plants used by man and is considered a panacea. It is used for its antibacterial, antiviral, antioxidant, antimalarial, anti-inflammatory, antidiabetic, cardiovascular, and antitumor effects [8]. In addition, it helps preserve food thanks to its antioxidant properties [9]. *S. lavandulifolia* is a widely distributed species in the Mediterranean basin, in eastern Spain, extending to the western Mediterranean: southeastern Spain and northwestern Africa (Morocco and Algeria) [10,11]. In traditional medicine, *S. lavandulifolia* is used to treat and prevent various diseases. It is used as a fungicidal, virucidal, and bactericidal agent [12]. Additionally, the aqueous extract of this herb is used as a popular hypoglycemic remedy for diabetic patients [13]. In Morocco, this plant grows spontaneously in various regions [12]. It is generally used as a spice in the cosmetics industry [14]. Its leaves are used in traditional medicine as an antiseptic, healing, choleretic, astringent, and hypoglycemic remedy [15,16], and its antibacterial and antidiabetic activities had also been confirmed [17]. Diabetes is a group of metabolic diseases in which hyperglycemia is the main characteristic. The high level of glucose in the blood generates, among other consequences, oxygen free radicals by auto-oxidation of glucose, which is associated with the pathogenesis of diabetes complications [18]. The persistence of the high content of glucose in the blood leads to its auto-oxidation. It will also induce the generation of free radicals, thus causing diabetic complications. For this, the control of hyperglycemia by use of natural products is a good way to prevent their appearance in diabetic patients. The main objective of this work is the characterization of phytochemicals and the evaluation of the effects: antioxidant, antimicrobial, anti-hyperglycaemic, and α-amylase enzyme inhibitor in vitro and in vivo of the aqueous extract of *S. lavandulifolia*, which is highly valued in traditional medicine as an aromatic plant with a medicinal character.

## 2. Results and Discussion

### 2.1. Quality Control of Plant Material

Plant material quality control results are shown in Table 1. The moisture content of the leaves of *S. lavandulifolia* is around 10.5%; this value is significantly lower than the limit value set at 12% [19]. The pH of our plant is acidic (5.26: acidophilic); its titratable acidity is 0.61% and contains 5.47% of mineral matter. The dosage of metallic trace elements present in *S. lavandulifolia* showed quite low levels of arsenic (0.0055 mg/g), chromium (0.0012 mg/g), antimony (0.001 mg/g), cadmium (0.0001 mg/g), copper (0.004 mg/g), and titanium (0.0049 mg/g) against a slightly high iron content (0.5099 mg/g) and an absence of lead. It is to be noted that these results are below the limit values for each MTE.

### 2.2. Phytochemical Screening

Phytochemical screening is a preliminary and of great importance step since it reveals the presence of constituents known for their various biological activities and medicinal properties. The results of the screening of the leaves of *S. lavandulifolia* are shown in Table 2. The phytochemical characterization tests carried out made it possible to highlight the richness of our plant in primary and secondary metabolites. Indeed, our plant is rich in proteins, lipids (sterols and triterpenes), sugars, and carbohydrates (oses and holosides) as well as polyphenols such as flavonoids (leucoanthocyanins, flavones), tannins, alkaloids, mucilages, and saponosides. These results agree with the literature, indeed, many researchers have confirmed the presence of sugars, polyphenols, and flavonoids in *S. lavandulifolia* [20,21,22].

### 2.3. Contents of Polyphenols, Flavonoids, and Condensed Tannins

**Figure 1:**
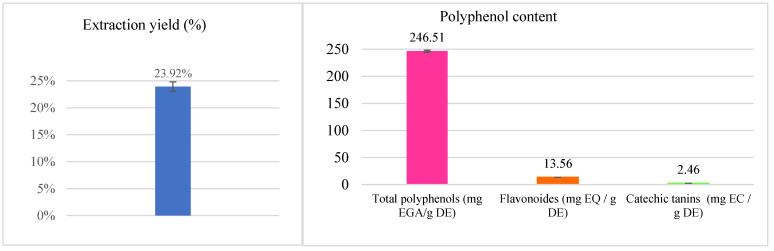
Extraction yield and contents of polyphenols, flavonoids and catechic tannins in the aqueous extract of *S. lavandulifolia*.

Polyphenols are secondary metabolites abundantly present in almost all species of aromatic and medicinal plants. These metabolites are essential for human nutrition due to their antioxidant properties and are beneficial to health [23]. The results of the various assays are grouped in Figure 1.

The extract of *S. lavandulifolia* is composed of 246.51 ± 1.69 mg EAG/g DE, 13.56 ± 0.08 mg EQ/g DE, and 2.46 ± 0.08 mg EC/g DE of total polyphenols, flavonoids, and condensed tannins, respectively. Our results are almost identical to those found by Boutahiri et al. (2021) in terms of total polyphenol content. They found that the aqueous extract of *S. lavandulifolia* obtained by decoction contains 252.67 ± 5.40 mg EAG/g DE [17]. Polyphenols are considered the most abundant group of secondary metabolites in plants, through which they defend themselves against predators and intrusions [24]. They are distinguished by the presence of hydroxyl groups, which enable them to be reactive by chelating metal ions and neutralizing free radicals through hydrogen atoms or electrons, thereby reducing their prooxidant activity [25,26]. Thanks to these characteristics, these molecules, endowed with preventive and curative effects of several diseases related to oxidative stress, have received great interest [27,28]. The family of polyphenols is divided into several classes of which flavonoids represent the majority (60%). They are responsible for the attractive colors of flowers, fruits, and leaves. They are more precisely called “nutraceuticals” thanks to their different pharmacological effects on the body. The advantages of being readily absorbed by the intestine, their ability to combat free radicals, and their hypoglycemic effect are what define flavonoids [29] as well as their hypoglycemic effect [30]. Tannins also have antioxidant power. Indeed, they block the formation of superoxide and the peroxidation of lipids [31].

Condensed tannins are oligomeric and polymeric byproducts of the biosynthesis of flavonoids [32]. Additionally, their capacity to scavenge free radicals is well known. They may not be present, but their quantity in the extracts is lower than that of the other bioactive compounds [33].

### 2.4. Chemical Composition of S. lavandulifolia Extract

**Figure 2:**
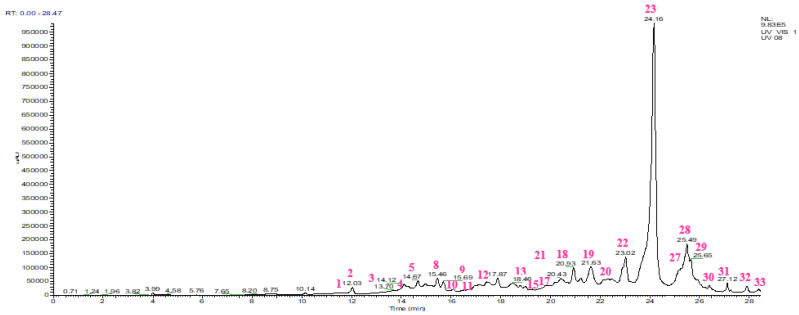
HPLC chromatogram of the aqueous extract of *Salvia lavandulifolia* Vahl.

The HPLC chromatogram and the chemical composition of the aqueous extract of *S. lavandulifolia* are shown in Figure 2 and Table 3. The below results show that our extract contains rosmarinic acid (37.03%), followed by quercetin rhamnose (7.84%), diosmetin-rutinoside (5.57%), catechin dimer (5.51%), gallocatechin (4.57%), luteolin (4.12%), caffeic acid (4.02%), carnosol (3.97%), catechin (2.62%), rhamnetin (1.84%), rutin (1.78%), azelaic acid (1.61%), ferulic acid (1.48%), and vanillic acid (1.46%) covering more than 80% of the overall composition of the extract. Several of these compounds are known for their important pharmacological activities. For example, rosmarinic acid, our major compound, is reputed to be antibacterial [34], antiviral [35], anti-inflammatory [36], anti-cancer [37], health-enhancing [38], and antioxidant [34,39]. Luteolin is known for its inhibitory effect on α-glucosidase and α-amylase enzymes [40]; it also has antioxidant, antimicrobial, anti-inflammatory, antidiabetic, neuroprotective, anticancer, and cardioprotective properties [41]. The antiradical activity of caffeic acid has been demonstrated [42]. Carnosol, for its part, is considered antioxidant, anticancer, anti-inflammatory, and antimicrobial [43]. Previous studies compiled by Claudia Musial and her collaborators indicated antitumor, antioxidant, anti-inflammatory, antimicrobial, antiviral, antidiabetic, antiobesity, and hypotensive effects related to catechins [44].

Our results agree with those of Salima Boutahiri et al., having identified rosmarinic acid, apigenin, luteolin, myricetin, herniarin, caffeic acid, protocatechuic acid, coumarin, cinnamic acid, vanillic acid, gallic acid, and chlorogenic acid in the aqueous extract of *S. lavandulifolia* leaves [17]. Salvador Cafligueral et al. revealed the presence of apigenin, luteolin, rosmarinic acid, quercetin-3-O-β-D-glucoside, luteolin-7-O-β-D-glucoside, luteolin-4′-O-glucoronide, luteolin-7-O-rutinoside, and other compounds such as nepetin and 5-Hydroxy-7, 4′-dimethoxyflavone in the soluble fraction of petroleum ether and chloroform extracts from the leaves of *S. lavandulifolia* [45].

### 2.5. Antioxidant Properties

The different antioxidant activities of Salvia lavandulifolia are shown in Table 4.

#### 2.5.1. Total Antioxidant Capacity

The PM (phosphomolybdate) test, such as CUPRAC (copper (II) ion reducing capacity) and FRAP (iron (III) reducing capacity), was selected to analyze the reduction capacity of *S. lavandulifolia* extract. This method involves the transfer of a single electron. In this system, the electron from the antioxidant that has been oxidized is transferred to the sub-strate, which prevents the oxidant from being reduced [46]. This assay quantifies the rate of reduction between antioxidant, oxidant, and molybdenum ligands and assesses the degree of reduction of Mo (VI) to Mo (V). It entails thermally generating auto-oxidation over an extended period of time at a high temperature. The advantage of this assay is to give a direct estimate of the antioxidant’s reducing capacity. Our extract showed a total antioxidant capacity of 527.03 ± 5.95 mg EQ AA/g ES as shown in Table 4. The latter is clearly high; this can be explained by the presence of luteolin known for its antioxidant capacity, chelator of transition metals [41].

#### 2.5.2. Free Radical Scavenging DPPH°

**Figure 3:**
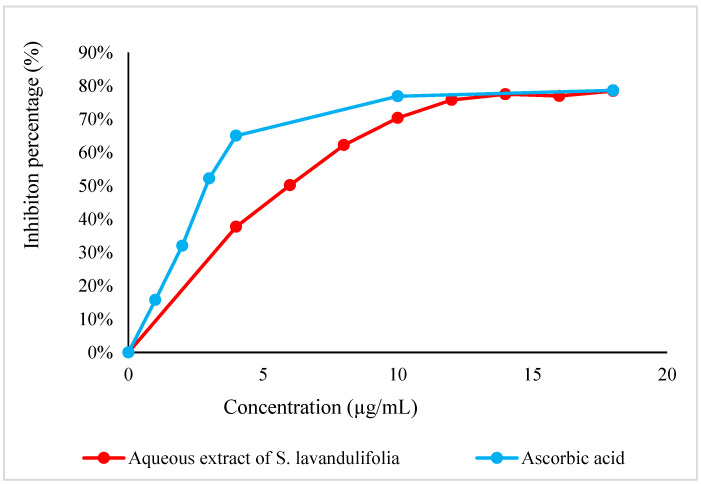
DPPH° radical scavenging curve by the aqueous extract of *S. lavandulifolia*.

DPPH° is a stable free radical that is frequently utilized to evaluate the antioxidant activity of natural compounds in a straightforward, quick, and accurate manner [47]. The results of this test, shown in Figure 3, indicate that *S. lavandulifolia* leaves have very significant antiradical activity, with an IC_50_ of 5.81 ± 0.23 µg/mL; however, its effect remains lower than that of acid ascorbic (IC_50_ = 3.06 ± 0.30 µg/mL). These results are consistent with the previous study by Pop et al., 2016 where the ability to scavenge DPPH° free radicals by the methanolic extract of *S. lavandulifolia* was demonstrated [48]. However, the antiradical property of this plant is linked to its richness in polyphenols. The latter is composed of hydroxyl groups that can neutralize free radicals [49]. Further UHLPC analysis shows the presence of caffeic acid and rutin potential responsible in minor part for this high capacity. As well as rosmarinic acid in particular, identified with a percentage of 37%, is reputed to have an important antiradical property [38,50].

### 2.6. Antimicrobial Activity

The antimicrobial activity of *S. lavandulifolia* extract was evaluated against strains of bacteria and fungi (Table 5). The results of the MIC values obtained show that the extract was more active against the strains tested. The lowest MIC found is 2.34 mg/mL against *Staphylococcus aureus*. The strains *Escherichia coli*, *Pseudomonas aeruginosa*, and *Candida krusei* are inhibited with an extract concentration of 18.75 mg/mL. In addition, the extract has also inhibited the following strains: *Enterobacter cloacae*, *Klebsiella pneumoniae*, *Staphylococcus epidermidis*, *Candida albicans*, *Candida tropicalis*, and *Saccharomyces cerevisiae* with an MIC = 37.5 mg/mL each. While *Escherichia coli* ESBL, *Proteus mirabilis*, *Streptococcus agalactiae* (B), *Aspergillus niger*, *Candida dubliniensis*, *Candida kyfer*, and *Candida parapsilosis* strains are inhibited starting from a concentration of 75 mg/mL. According to the MBC/MIC ratio, the extract of *S. lavandulifolia* reported a bactericidal effect against *Proteus mirabilis* and fungicidal against strains *Aspergillus niger*, *Candida albicans*, *Candida tropicalis*, *Saccharomyces cerevisiae*, and *Candida krusei*. Based on these findings, *Salvia lavandulifolia* extract could potentially be used as natural preservatives in foods against well-known causative agents of foodborne illnesses such as *S. aureus* and *E. coli* [17]. In this work, the inhibitory activities of this extract are probably due mainly to the action of the majority compounds in this extract: rosmarinic acid, quercetin rhamnose, diosmetin-rutinoside, and catechin dimer [21]. The antimicrobial activity of rosmarinic acid against various bacterial and fungi strains has been described by a number of authors. Additionally, a study was carried out by Giner and his associates [51] on the combination of hydroalcoholic extracts of *S. lavandulifolia*, *S. rosmarinus*, and *T. mastichina*. With an MIC value of 12.8 mg/mL, they demonstrated that it is effective against *E. coli* and *E. aerogenes*; these data are consistent with our findings.

### 2.7. Toxicity

Traditional MAP-based treatments can induce toxicity problems leading to treatment failures. For this, we conducted pharmacological tests in vivo after first assessing the toxicity of the aqueous extract of *S. lavandulifolia*. This experiment aims to demonstrate that the therapeutic doses of *S. lavandulifolia* extract (0.5 g/kg, 1 g/kg, or 2 g/kg) do not cause short-term toxicity in healthy mice. According to the test’s findings, the extract is not toxic even at a dose of 2 g/kg. Throughout the entire follow-up period, it did not result in any toxicity symptoms (such as diarrhea, vomiting, abnormal mobility, etc.) or fatalities. According to Perry et al. (2001), long-term use of *S. lavandulifolia* as a food flavoring agent did not induce adverse effects [52].

### 2.8. Antihyperglycemic Effect

**Figure 4:**
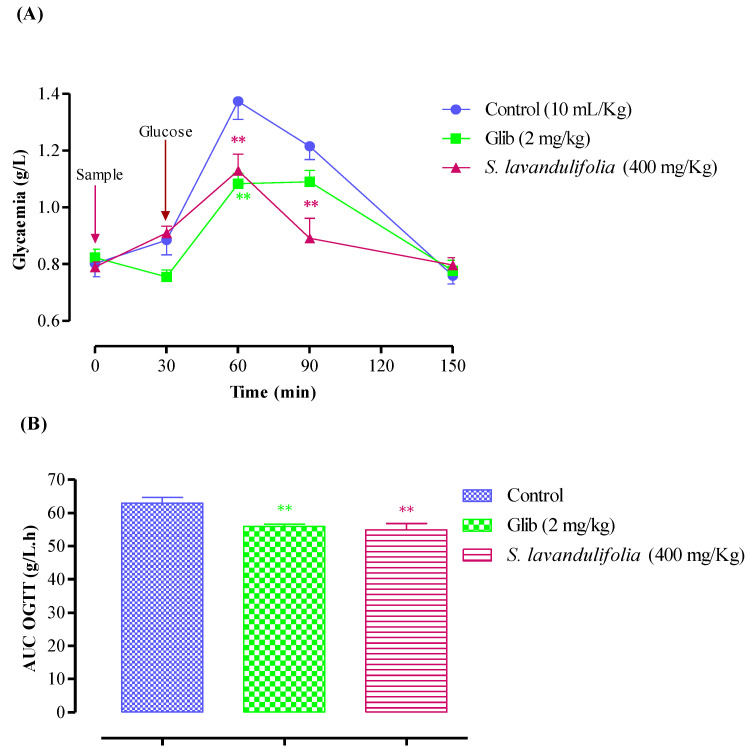
Postprandial blood glucose (**A**) and area under the postprandial curve (**B**) in normal rats after administration of the products tested (*S. lavandulifolia* extract and glibenclamide), ** *p* < 0.01; compared to the control.

In normal rats, oral administration of the *S. lavandulifolia* aqueous extract at 400 mg/Kg 30 min before the glucose overload significantly reduced post-prandial hyperglycemia at 60 min (*p* < 0.01, 1.13 ± 0.14 g/L) and at 90 min (*p* < 0.01, 0.89 ± 0.17 g/L). In a similar manner, glibenclamide significantly reduced postprandial hyperglycemia for two hours after glucose overload at 60 min (*p* < 0.01; 1.08 ± 0.09 g/L) and 90 min (*p* < 0.05; 1.09 ± 0.10 g/L). In comparison to pretreated distilled water, there was no discernible difference in blood glucose levels between the two groups at 150 min; (0.76 ± 0.07 g/L) at 150 min (1.22 ± 0.11 g/L) at 90 min, and at 60 min (1.37 ± 0.16 g/L) (Figure 4A). In rats treated with the aqueous extract of *S. lavandulifolia* (54.84 ± 4.88 g/L/h) as opposed to rats treated with distilled water (62.91 ± 4.32 g/L/h), the area under the curve (AUC glucose) is significantly lower in the former group (*p* < 0.01) (Figure 4B). It is also less than the positive control (glibenclamide), which is significantly less (55.95 ± 1.69 g/L/h; *p* < 0.01) than the rats given distilled water. Additionally, it is less than the positive control (glibenclamide), which has a significantly lower concentration (55.95 ± 1.69 g/L/h; *p* < 0.01) than rats given distilled water as a treatment.

These results are consistent with those of Jimenez et al. from 1986, who demonstrated that administration of an aqueous extract of *S. lavandulifolia* 60 min before glucose over-load induced marked antihyperglycemic activity, compared to administration of glucose alone. This finding suggests that intestinal glucose uptake may be a key factor that may explain this activity [53]. The majority of Salvia species used in traditional medicine to treat diabetes frequently work by boosting insulin secretion, boosting adipose tissue and skeletal muscle glucose uptake, and reducing intestinal glucose absorption and hepatic glycogenolysis [54]. In a different study, the hypoglycemic activity of *S. lavandulifolia* was attributed to its capacity to promote glucose uptake in peripheral tissues (muscle and adipose tissue), which results in the return of blood sugar to normal levels [54]. Studies on this plant’s aqueous extract have similarly demonstrated its hypoglycemic effects by raising pancreatic insulin secretion and peripheral glucose uptake [54,55].

### 2.9. Pancreatic α-Amylase Inhibitory Effect

#### 2.9.1. In Vitro Test

**Figure 5:**
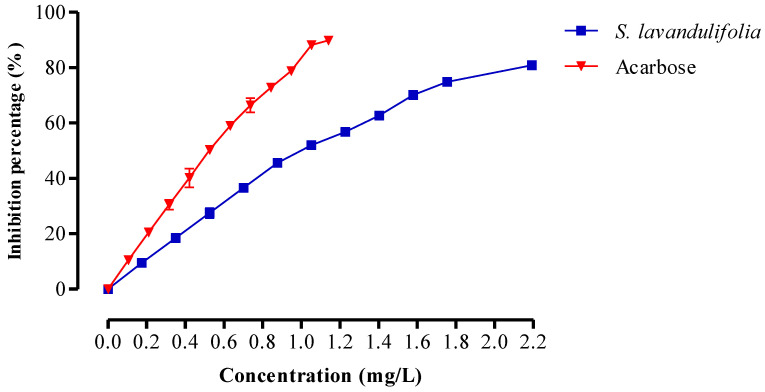
Inhibitory effect on α-amylase activity by aqueous extract of *S. lavandulifolia* and acarbose, in vitro.

An enzyme called pancreatic α-amylase breaks down polysaccharides (such as starch and glycogen) into disaccharides. Figure 5 depicts the impact of the *S. lavandulifolia* aqueous extract on the in vitro activity of this enzyme. In fact, our extract significantly inhibited the activity, with an IC_50_ of 0.99 ± 0.00 mg/mL compared to IC_50_ = 0.52 ± 0.01 mg/mL for acarbose. Inhibiting α-amylase activity is one of the most efficient therapeutic strategies to manage postprandial hyperglycemia in diabetic patients [56]. The concentration of fibers in *S. lavandulifolia* and the presence of inhibitors on these fibers reduce the accessibility of starch to the enzyme, decreasing the activity of α-amylase. These are just a few of the factors that contribute to this inhibition [57].

#### 2.9.2. In Vivo Test

Oral administration of aqueous extract of *S. lavandulifolia* at C = 400 mg/kg 30 min before starch overload in normal rats significantly reduced postprandial hyperglycemia at 60 min (*p* < 0.001, 0.90 ± 0.06 g/L), at 90 min (*p* < 0.001; 0.83 ± 0.03 g/L), and at 150 min (*p* < 0.001; 0.84 ± 0.02). 

**Figure 6:**
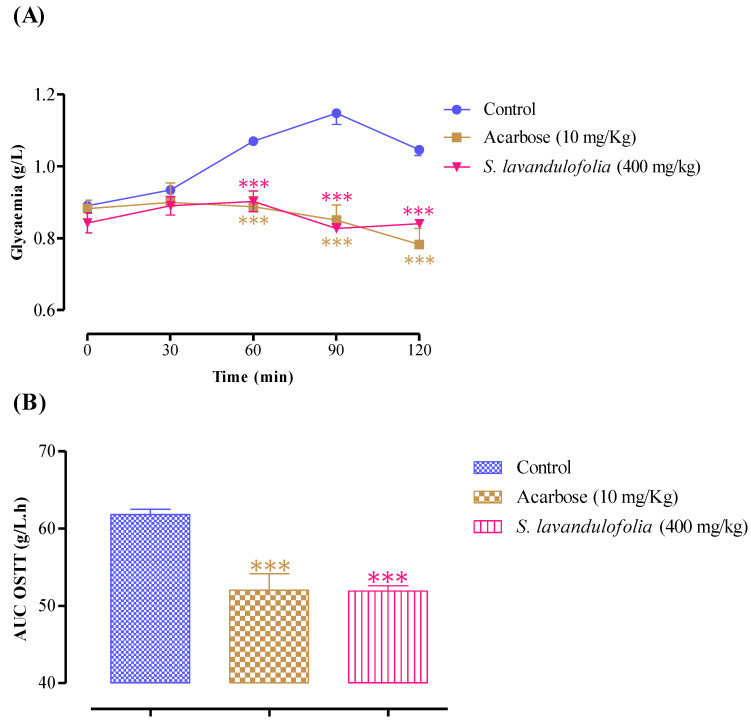
Effect of *S. lavandulifolia* extract and acarbose on the variation in postprandial glycaemia in normal rats (**A**), with a representation in the form of areas under the curves (**B**), *** *p* < 0.001: relative to the control.

Similar to this, acarbose significantly reduced postprandial hyperglycemia during the two hours that followed starch overload at 60 min (*p* < 0.001, 0.89 ± 0.06 g/L), at 90 min (*p* < 0.001, 0.85 ± 0.08 g/L), and at 150 min (*p* < 0.001, 0.78 ± 0.09 g/L) (Figure 6A). Rats pretreated with distilled water only recorded remarkable hyperglycemia, unlike the first and second groups, at 60 min (1.07 ± 0.02 g/L), at 90 min (1.15 ± 0. 07 g/L), and 150 min (1.05 ± 0.04 g/L). In addition, the area under the curve (AUC glucose) was significantly lower (*p* < 0.001) in rats treated with plant extracts (51.94 ± 1.29 g/L/h) than those treated with acarbose (52.05 ± 4.27 g/L/h) and distilled water (61.82 ± 1.53 g/L/h) (Figure 6B). As mentioned above, *S. lavandulifolia* is rich in polyphenols and flavonoids. Moreover, phytochemical analysis of this aqueous extract showed that it contains apigenin, rosmarinic acid, and luteolin, responsible for the inhibitory effect of pancreatic α-amylase [40,58,59,60], hence, the drop in blood sugar levels. 

## 3. Material and Methods 

### 3.1. Plant Material

*S. lavandulifolia* is a species of the genus Salvia, family Lamiaceae, and order Lamiales. It is commonly called lavender sage or Spanish sage. Our drug was harvested in the rural town of Ouled Ali in the province of Boulemane-Morocco in May 2020 (Table 6) and was dried in the open air and protected from light for two weeks.

### 3.2. Quality Control of Plant Material of S. Lavandulifolia

#### 3.2.1. Humidity Level

A quantity of 5 g of dry plants was put in Petri dishes and left in the oven at a temperature of 100 ± 5 °C for 24 h [61,62].

$$\mathrm{HL}\;\left(\%\right)=\frac{\left(m_{1}-m_{2}\right)}{m_{1}}\times 100$$

m_1_: initial mass of the plant before drying in the oven (g),

m_2_: final mass of the plant after drying in the oven (g).

#### 3.2.2. pH Determination

A mass (5 g) of the sample was combined with 500 mL of distilled water. A stirrer and magnetic bar were used to stir the mixture for 5 min at room temperature. After that, the mixture was filtered. The pH was determined using an STPURE electrode-equipped benchtop pH meter, the Ohaus Starter 3100 [63]. The pH reading was then taken by placing the electrode of the pH meter into a volume of filtrate.

#### 3.2.3. Determination of Titratable Acidity

Ten grams of herbal drug powder was extracted by use of 100 mL of boiling water for 15 min. Following filtration, the mixture was combined with 20 mL of distilled water from 10 mL of the filtrate. Following the addition of a few drops of phenolphthalein, the titration procedure was continued using a solution of NaOH (0.01 N) until a persistent pink color was achieved. The volume of NaOH poured up to the equivalence point is converted into equivalent citric acid using the following formula [64].

$$\mathrm{Total}\;\mathrm{acidity}=\frac{\mathrm{dilution}\;\mathrm{factor}\;\times \;\mathrm{weight}\;\mathrm{of}\;\mathrm{eq.Acid}\;\times \mathrm{normality}\;\mathrm{of}\;\mathrm{NaOH}\;\times \;\mathrm{titration}\;\mathrm{vol.(mL)}\;}{\mathrm{sample}\;\mathrm{mass}\;\left(g\right)}$$

#### 3.2.4. Ash Content

The organic matter content is determined by calculating the difference in weight before and after calcination. The latter consists of passing 2 g of ground sample in a muffle furnace at a temperature of 550 °C, up to the total destruction of any carbonaceous particles (light gray or whitish color) [65]. The organic matter content is calculated using the following formula:

$$\mathrm{OM}\%=\frac{m_{1}-m_{2}}{\mathrm{TS}}\times 100$$

OM%: Organic matter;

m_1_: Pre-calcination capsule and sample mass;

m_2_: Post-calcination capsule and sample mass; 

TS: Test sample.

The ash content was calculated as follows: MM% = 100 − MO%

#### 3.2.5. Dosage of Metallic Trace Elements (MTE) by ICP-AES

For the determination of MTE contents (As, Cr, Sb, Pb, Cd, Fe, Cu, and Ti), the mineralization protocol with aqua regia (HNO_3_ + 3HCl) was adopted. The method consists of mixing 0.1 g of crushed plant material with 3 mL of aqua regia and heating it under reflux (200 °C) for two hours. After cooling and settling, the supernatant is recovered and filtered on a membrane (0.45 µm) and then made up to 15 mL with water. Notably, the inductively coupled plasma atomic emission spectrometer (ICP-AES) was used to measure the MTE concentrations. [66].

### 3.3. Preparation of the Aqueous Extract of Salvia lavandulifolia

Briefly, 30 g of the crushed plant was introduced into a reflux assembly with 750 mL of distilled water. The mixture was heated at 80 °C with stirring for one hour and filtered [62]. The extract was dried in an oven at 70 °C overnight in a silicone mold and then collected in amber glass bottles.

$$Y\;\left(\%\right)=\frac{m_{2}}{m_{1}}\times 100$$

Y: yield; m_2_: mass of the extract; m_1_: mass of the crushed plant.

### 3.4. Phytochemical Screening

Phytochemical screening tests are qualitative tests that consist of detecting the different families of compounds present in plant material. They are based on coloring, precipitation, or complexation reactions [67,68].

### 3.5. Phenolic Compounds

#### 3.5.1. Determination of Total Polyphenol Content

The Singleton et al. protocol was utilized to determine total phenolic content (TPC), with a few minor modifications [69]. Quantities of 15 µL (C = 25 mg/mL) of extract, 1.5 mL of Folin Ciocalteux’s reagent (10%), and 1.5 mL of Na_2_CO_3_ (7.5%) were introduced, respectively, into 50 mL volumetric flasks and supplemented with distilled water. The mixture was blended and incubated at room temperature for 40 min in the dark. The concentration of phenolic compounds in the *S. lavandulifolia* extract was expressed in equivalents of gallic acid (EGA), and the absorbance at a wavelength of 760 nm was measured. 

#### 3.5.2. Determination of Flavonoid Content

To test tubes, 30 µL (C = 25 mg/mL) of extract, 2 mL of distilled water, and 10 µL of aluminum chloride prepared in methanol (10%, m/V) were added. Pure methanol was used to dilute the mixture to a total volume of 5 mL. The solutions were mixed and incubated in the dark for 30 min. At 433 nm, absorbance was measured, and flavonoid concentration was expressed as quercetin equivalents (QE) [70].

#### 3.5.3. Determination of Condensed Tannin Content

100 µL (C = 25 mg/mL) of extract, 3 mL of vanillin methanolic solution (4%, m/V), and 1.5 mL of HCl (37%) were added in test tubes. The contents of the tubes were mixed and incubated at room temperature for 20 min in the dark. At 499 nm, absorbance was measured, and condensed tannin concentration was expressed in CE catechin equivalent [71].

### 3.6. Identification of the Chemical Composition by HPLC-MS-ESI

The HPLC-MS analysis was conducted by the Dionex UltiMate 3000 ULC/HPLC system coupled to an Exactive mass spectrometer with an ESI ionization source and an orbitrap analyzer. A volume of 10 μL of extract dissolved in distilled water (C = 100 μg/mL) was injected into a C18 column with 100 mm long, 2.1 mm in diameter, and with 1.7 µm pores. The temperature was programmed at 30 °C, while the flow rate was 0.45 mL/min. The mobile phase contained two solvents: solvent A (Water + formic acid (0.1%), *v*/*v*) and solvent B (Acetonitrile + formic acid (0.1%), *v*/*v*). The established elution gradient was “A+B” [98:2] (0–19 min), “A+B” [70:30] (20–24 min), “A+B” [5:95] (25 min), and “A+B” [98:2] (26–30 min). The detection was carried out using a diode array detector by scanning in the wavelength range of 280–360 nm, as well as by the spectrometer of mass (Exactive) after negative ionization. Data were acquired using MASS LYNX software(Version 4.2). The molecules were identified based on retention time, mass spectrum, molecular weight, and by comparison with standards (injected under the same conditions as the extract): caffeic acid, coumaric acid, ferulic acid, gallic acid, rosmarinic acid, sinapic acid, syringic acid, tannic acid, trans-cinnamic acid, vanillic acid, apigenin, catechin, coumarin, kaempferol, luteolin, myricetin, and rutin.

### 3.7. Antioxidant Activity Test

#### 3.7.1. Total Antioxidant Capacity

The test sample was mixed with 1 mL of ammonium molybdate (4 mM), 1 mL of sodium phosphate (28 mM), and 1 mL of sulfuric acid (0.6 M) in test tubes. The tubes’ contents were combined and incubated at 95 °C for ninety minutes before being normalized for 20 to 30 min at room temperature. At 695 nm, the measurement was made. The results were expressed in milligram equivalents of ascorbic acid per gram of dry extract (mg EAA/g DE), with ascorbic acid serving as the control [72].

#### 3.7.2. 2,2′-Diphenyl-1-Picryl Hydroxyl Test

An increasing volume of extract was put into test tubes, and ethanol was added to reach a total volume of 200 µL. A quantity of 2.8 mL of ethanolic solution of DPPH° (24 µg/mL, m/V) was added to the mixture and left to incubate for 30 min in the dark. Notably, UV–Vis absorbance was measured at 515 nm [73].

$${\%\;}_{\mathrm{inhibition}}=\frac{A_{C}-A_{S}}{A_{C}}\times 100\;;\;{\mathrm{EC}}_{50}=\frac{{\mathrm{IC}}_{50}}{C_{\mathrm{DPPH}}}$$

A_C_: Absorbance of the negative control, A_S_: Absorbance of the sample, IC_50_: Inhibiting concentration 50% of DPPH° radicals (mg/mL), C_DPPH_: Concentration of DPPH° (mg/mL).

EC_50_ effective concentration was used to calculate the antiradical potency. The higher the ARP, the more effective the antioxidant [74].

$$\mathrm{ARP}=\frac{100}{{\mathrm{EC}}_{50}}$$

EC_50_: Effective concentration of the sample.

### 3.8. Antimicrobial Activity

#### 3.8.1. Preparation of Microbial Suspensions

The antimicrobial activity of *S. lavandulifolia* aqueous extract was tested against nine bacteria and fungi (Table 7). These pathogenic microorganisms are frequently encountered in many infections, causing clinical and therapeutic issues. All strains were first frozen in 20% glycerol stock at −80 °C and then regenerated on Mueller–Hinton or Sabouraud broths and finally subcultured.

#### 3.8.2. Determination of MIC and MBC/MFC

The minimum inhibitory concentration (MIC) was determined by use of 96-well microplates using the reference method of microdilution [75]. From an extract stock solution prepared in a 30:70 ethanol/distilled water mixture, a series of dilutions were carried out to obtain various concentrations ranging from 75 to 4.6875 mg/mL, with a final volume of 100.00 μL in Mueller–Hinton (MH) broth for the bacterial strain and Sabouraud broth for the fungal strain. Following that, 100 µL of microbial suspension (inoculum) with a final concentration of 10^6^ CFU/mL for bacteria or 10^4^ CFU/mL for fungi was added to the different wells (1 to 11), with the 11th and 12th wells serving as growth and sterility controls, respectively. After incubating for 24 h at 37 °C, 10 μL of resazurin was added to each well as a microbial growth indicator before reincubating for two hours at 37 °C. The change in color from purplish blue to bright pink revealed growth. The MIC is defined as the lowest concentration that prevents resazurin from changing color. To determine the MBC or MFC, 10 µL was taken from each well where no growth could be seen and put on MH agar for bacterial growth or Sabouraud for fungal growth for 24 h at 37 °C. After incubation, MBC/MFC is determined as the lowest concentration inhibiting colony formation on solid agar medium [76]. To evaluate antimicrobial potency, the MBC/MIC or MFC/MIC ratio can be calculated. Indeed, if the ratio is less than 4, the extract is bactericidal/fungicidal; if it is greater than 4, the sample is bacteriostatic/fungistatic [77].

### 3.9. Animals

*Wistar rats* (200–250 g) and albino mice (25–35 g) were used in this study and were reared under ideal conditions photoperiods of 12 h light/12 h dark and 22 ± 2 °C) with free access to water and food. This test was carried out in accordance with the Organization for Economic Cooperation and Development’s guidelines (OECD) [78].

### 3.10. Acute Toxicity

A quantity of 24 albino mice (20–35 g) on an empty stomach (14 h) were randomly distributed into four groups (*n* = 6; ♂/♀ = 1). The control group received distilled water (10 mL/kg) and the treated groups received the doses: 0.5 g/kg, 1 g/kg, and 2 g/kg. When the test first began, the mice were weighed. Immediately afterwards, they received a single dose of the test extract, orally. Then, they were continuously monitored for 10 h to report any apparent signs of toxicity. For the remaining 14 days, the mice were kept under daily surveillance for further clinical or behavioral signs of toxicity. This test was performed in accordance with the guidelines of the Organization for Economic Co-operation and Development (OECD) [78].

### 3.11. Antihyperglycemic Effect

The oral glucose tolerance test was performed to evaluate the antihyperglycemic (postprandial glucose) effect in vivo [79]. Normal rats were divided into three groups (*n* = 6; ♂/♀ = 1): control group: distilled water (10 mL/kg) and test groups: normal rats force-fed with the extract (400 mg/kg) or glibenclamide (2 mg/kg). First, blood glucose was measured at t_0_ just before administration of the test product (distilled water, aqueous extracts, or glibenclamide). A total of 30 min later, another measurement of glycemia was carried out then the rats were overloaded with D-glucose (2 g/kg). Subsequently, the variation in blood glucose was measured every half hour up to 90 min and then after one hour.

### 3.12. Pancreatic α-Amylase Inhibitory Effect

#### 3.12.1. In Vitro Test

The Nageswara Rao Thalapaneni et al. [80] method with some modifications was used to examine the inhibitory effect of *S. lavandulifolia* aqueous extract on pancreatic-amylase enzymatic activity. Increasing volumes of *S. lavandulifolia* extract/acarbose were added to test tubes, which were then filled to a capacity of 200 µL with distilled water.

The tubes were then filled with 200 µL of pancreatic α-amylase solution (61.33 U/mL) and 200 µL of phosphate buffer (PB) solution (0.02 M; pH = 6.9), with the exception of the correction series, where the PB solution was used in its place. A time of 10 min at 37 °C were spent pre-incubating the tubes.

The tubes were then re-incubated for 15 min at 37 °C with 200 µL of a 0.5% starch solution. After adding 600 µL of DNSA (2.5%) to halt the enzymatic reaction, the tubes were heated in a boiling water bath for 8 min. The tubes were placed in an ice bath to stop the reaction, and then 10 mL of diluted water was added to each tube. In order to compare the absorbance at 540 nm to the correction series, a spectrophotometer was used. The percentage inhibition was calculated using the equation shown below:

$$\mathrm{Inhibitory}\;\mathrm{activity}\;\mathrm{percentage}=\frac{\left({\mathrm{Ab}}_{\mathrm{Control}}-{\mathrm{Ab}}_{\mathrm{Blank}})-({\mathrm{Ab}}_{\mathrm{Test}}-{\mathrm{Ab}}_{\mathrm{Blank}\;\mathrm{of}\;\mathrm{the}\;\mathrm{corresponding}\;\mathrm{tube}\;}\right)}{\left({\mathrm{Ab}}_{\mathrm{Control}}-{\mathrm{Ab}}_{\mathrm{Blank}}\right)}\times 100$$

with:

Ab_Control_: absorption of enzyme activity without inhibitor; 

Ab_Test_: Absorption of enzymatic activity in the presence of the extract or acarbose; 

Ab_Blank/Blank of the corresponding tube_: a series of corrections is carried out in parallel with each series where the enzyme is replaced by the PB.

#### 3.12.2. In Vivo Test

The purpose of this test is to confirm the enzymatic activity of pancreatic-amylase-inhibitory effect of *S. lavandulifolia* aqueous extract in vivo in normal rats by taking into account the effect of intestinal lumen on the extract’s inhibitory effect. Normal fasting rats (180–250 g, 14 h) were divided into three groups (*n* = 6; ♂/♀ = 1): the control group was given distilled water (10 mL/kg), while the treated groups were given the extract (400 mg/kg) or acarbose (10 mg/kg). After measuring blood glucose, an adequate volume of the extract/distilled water/acarbose was administered (t_0_) to begin the oral starch tolerance test. A second measurement of glycemia was taken 30 min later (t_1_), and the rats were then overloaded with starch (3 g/kg). The variation in glycemia was measured at t_2_ = 60 min, t_3_ = 90, and t_4_ = 120 min.

### 3.13. Statistical Analysis

The results were statistically analyzed using ANOVA (one-way analysis of variance with Tukey’s post hoc test), and they are shown as means and standard deviation. Statistics were considered to be significant at *p*-values of *p* < 0.05, *p* < 0.01, and *p* < 0.00.

## 4. Conclusions

Aromatic and medicinal plants are a precious gift of nature for human beings; they have been used since antiquity in food, beverages, traditional medicine, and cosmetics. *Salvia lavandulifolia* Vahl is a widespread plant in Morocco; its leaves are often used for their high content of essential oil whose virtues are well known. The extracts, on the other hand, are very little studied by researchers, and their properties are found most of the time in articles—ethnopharmacological surveys or some very old ones. The aqueous extract of the leaves of *S. lavandulifolia* harvested in Ouled Ali-Boulemane-Morocco is very rich in polyphenols; it recorded a remarkable total antioxidant capacity and antiradical power. It is also considered bactericidal against *Proteus mirabilis*, fungicidal against *Aspergillus niger*, *Candida albicans*, *Candida tropicalis*, and *Saccharomyces cerevisiae*, and fungistatic against *Candida krusei*. Administered 30 min before an acute oral administration of glucose, our extract significantly lowered hyperglycemia in vivo in rats. Thanks to its content of rosmarinic acid, apigenin, and luteolin, it essentially could also inhibit the activity of the enzyme α-amylase in vitro and in vivo.

## Figures and Tables

**Table 1:** Humidity level, pH, ash, and heavy metals present in *S. lavandulifolia*.

**S. L**	**Humidity Level**		**pH**		**Titratable Acidity**		**Mineral Matter**	
	10.47 ± 0.05		5.26		0.61 ± 0.00		5.47 ± 0.60	
	**Concentration of some MTE (mg/g)**							
	**As**	**Cr**	**Sb**	**Pb**	**Cd**	**Fe**	**Cu**	**Ti**
	0.0055	0.0012	0.0010	Undetectable	0.0001	0.5099	0.0040	0.0049
**LV (mg/L)**	0.05	0.05	0.005	0.05	0.005	20	1	–

LV: limit value.

**Table 2:** Chemical families present in *S. lavandulifolia*.

	Chemical Group		*S. lavandulifolia*
**Secondary Metabolites**	Tannins		+
	Catechic tannins		+
	Gallic tannins		+
	Flavonoids		+
	Cyanidin reaction		Flavones
	Leucoanthocyanins		+
	Saponosides		+
	Alkaloids		-
	Reducing Compounds		+
	Monosaccharides and holosides		+
	Mucilages		+
	Sterols and triterpenes		+
**Primary metabolites**	Polysaccharide		glycogen
	Reducing sugar (glycose, fructose)		+
	Protein	Biuret reaction	+
		Xanthoprotein reaction	+
	Lipids (Lieberman Burchard reaction)		+

**Table 3:** Chemical composition of the aqueous extract of *Salvia lavandulifolia* Vahl.

N°	RT (min)	Area (%)	(*m*/*z*) [M − H]^+^	MW	Identified Compound	Molecular Formula
1	11.58	0.65	197/391	198	Syringic acid	C_9_H_10_O_5_
2	12.03	1.07	447	448	Quercetin 3-O-rhamnoside	C_21_H_20_O_11_
3	13.7	1.33	293	294	Cynamil p-methoxy cinnamate	C_19_H_18_O_3_
4	14.28	1.39	431	432	Apigenin 7-O-glucoside	C_21_H_20_O_10_
5	14.67	1.18	503	504	6-caffeoylsucrose	C_21_H_28_O_14_
6	14.97	1.48	387	194	Ferulic acid	C_10_H_10_O_4_
7	15.14	0.92	463	464	Qercetin-3-O-β-D-glucoside	C_21_H_20_O_12_
8	15.46	1.46	167/335	168	Vanillic acid	C_8_H_8_O_4_
9	15.69	1.05	341	342	1-caffeoyl-beta-D-glucose	C_15_H_18_O_9_
10	16.09	0.54	181	180	Trans-Caffeic acid	C_9_H_8_O_4_
11	16.55	0.54	325	326	Trans-p-coumaric acid-4-O-β-D-glucopyranoside	C_15_H_18_O_8_
12	17.45	4.12	387	388	Luteolin	C_15_H_10_O_6_
13	18.46	4.02	179	180	Caffeic acid	C_9_H_8_O_4_
14	18.79	0.66	355	354	Chlorogenic acid	C_16_H_18_O_9_
15	18.99	0.72	539	540	Yunnaneic acid D	C_27_H_24_O_12_
16	19.27	1.84	315	316	Rhamnetin	C_16_H_12_O_7_
17	19.84	1.61	375	188/375	Azelaic acid	C_9_H_16_O_4_
18	20.93	5.51	575	576	Catechin dimer	C_30_H_24_O_12_
19	21.63	7.84	447	448	Quercetin rhamnose	C_21_H_20_O_11_
20	22.31	1.38	417	418	Luteolin pentose	C_20_H_18_O_10_
21	22.47	1.78	611	610	Rutin	C_27_H_30_O_16_
22	23.02	4.57	307	306	Gallocatechin	C_15_H_14_O_7_
23	24.16	37.03	359	360	Rosmarinic acid	C_18_H_16_O_8_
24	24.5	0.68	473	474	Dicaffeoyl tartrate	C_22_H_18_O_12_
25	24.8	2.62	291	290	Catechin	C_15_H_14_O_6_
26	25.16	0.99	183	184	Methylgallate	C_8_H_8_O_5_
27	25.25	1.15	345	346	Rosmanol	C_20_H_26_O_5_
28	25.49	5.57	607	608	Diosmetin-rutinoside	C_28_H_32_O_15_
29	25.65	3.97	329	330	Carnosol	C_20_H_26_O_4_
30	26.39	0.66	285	286	Kaempferol	C_15_H_10_O_6_
31	27.12	0.49	313	314	Cirsimaritine	C_17_H_14_O_6_
32	27.9	0.73	345	346	Metoxy-carnosic acid	C_21_H_30_O_4_
33	28.39	0.45	191	192	Quinic acid	C_7_H_12_O_6_
	Total	100%				

**Table 4:** PM and DPPH° antioxidant activities of the aqueous extract of *Salvia lavandulifolia* Vahl.

	TAC (mg EAA/g Es)	DPPH°		
		IC_50_ (µg/mL)	EC_50_	ARP
**Aqueous Extract of S. l.**	527.03 ± 5.95	5.81 ± 0.23	0.24 ± 0.01	413.43 ± 16.45

**Table 5:** MIC and MBC/MFC of the microbial strains studied.

Strains	MIC (mg/mL)	MBC/MFC (mg/mL)	Report
***Enterobacter cloacae***	37.5	>75	-
***Escherichia coli*** **BLSE**	75	>75	-
***Escherichia coli*** **sauvage**	18.75	>75	-
***Klebsiella pneumoniae***	37.5	>75	-
***Proteus mirabilis***	75	75	1
***Pseudomonas aeruginosa***	18.75	>75	-
***Staphylococcus aureus*** **BLACT**	2.34	>75	-
***Staphylococcus epidermidis***	37.5	>75	-
***Streptococcus agalactiae*** **(B)**	75	>75	-
***Aspergillus niger***	75	75	1
***Candida albicans***	37.5	75	2
***Candida dubliniensis***	75	>75	-
***Candida kyfer***	>75	>75	-
***Candida krusei***	18.75	75	4
***Candida parapsilosis***	75	>75	-
***Candida tropicalis***	37.5	75	2
***Saccharomyces cerevisiae***	37.5	75	2

**Table 6:** Information about the studied plant.

Plant Species	Family	Voucher Number	Used Organ	Harvest Region	Geographical Coordinates	Harvest Date
*S. lavandulifolia*	Lamiaceae	RAB111857	Leaves	Ouled Ali	33°28′41″ N 3°59′35″ O	May 2020

**Table 7:** List of bacterial and fungal strains tested.

Bacterial Strains	Code	Fungal Strains	Code
*Enterobacter cloacae*	02EV317	*Aspergillus niger*	AspN
*Escherichia coli* BLSE	2DT2057	*Candida albicans*	Ca
*Escherichia coli sauvage*	3DT1938	*Candida dubliniensis*	Cd
*Klebsiella pneumoniae*	3DT1823	*Candida kyfer*	Cky
*Proteus mirabilis*	2DS5461	*Candida krusei*	Ckr
*Pseudomonas aeruginosa*	2DT2138	*Candida parapsilosis*	Cpa
*Staphylococcus aureus* BLACT	4IH2510	*Candida tropicalis*	Ct
*Staphylococcus epidermidis*	5994	*Saccharomyces cerevisiae*	Sacc
*Streptococcus agalactiae* (B)	7DT1887		

## Data Availability

Not applicable.
